# General peroxidase activity of a parallel G-quadruplex-hemin DNAzyme formed by Pu39WT - a mixed G-quadruplex forming sequence in the Bcl-2 P1 promoter

**DOI:** 10.1186/1752-153X-8-43

**Published:** 2014-07-01

**Authors:** Bo Liu, Da Li, Hong Shang

**Affiliations:** 1 Department of Laboratory Medicine, the First Hospital of China Medical University, Shenyang 110001, China

**Keywords:** G-quadruplex, DNAzyme, Peroxidase activity, Bcl-2 promoter, Pu39WT

## Abstract

**Background:**

A 39-base-pair sequence (Pu39WT) located 58 to 19 base pairs upstream of the Bcl-2 P1 promoter has been implicated in the formation of an intramolecular mixed G-quadruplex structure and is believed to play a major role in the regulation of bcl-2 transcription. However, an extensive functional exploration requires further investigation. To further exploit the structure–function relationship of the Pu39WT-hemin DNAzyme, the secondary structure and peroxidase activity of the Pu39WT-hemin complex were investigated.

**Results:**

Experimental results showed that when Pu39WT was incubated with hemin, it formed a uniparallel G-quadruplex-hemin complex in K^+^ or Na^+^ solution, rather than a mixed hybrid without bound hemin. Also, Pu39WT-hemin showed peroxidase activity (ABTS^2−^) in the presence of H_2_O_2_ to produce the colored radical anion (ABTS^•-^), which could then be used to determine the parameters governing the catalytic efficiency and reveal the peroxidase activity of the Pu39WT-hemin DNAzyme.

**Conclusions:**

These results demonstrate the general peroxidase activity of Pu39WT-hemin DNAzyme, which is an intramolecular parallel G-quadruplex structure. This peroxidase activity of hemin complexed with the G-quadruplex-forming sequence in the Bcl-2 gene promoter may imply a potential mechanism of hemin-mediated cellular injury.

## Background

A 39-base-pair sequence, dAG_4_CG_3_CGCG_3_AG_2_A_2_G_5_CG_3_AGCG_4_CTG, named Pu39WT, located 58 to 19 base pairs upstream of the Bcl-2 P1 promoter, has been implicated in the formation of a mixed intramolecular G-quadruplex structure [1-3]. As a putative promoter, the G-quadruplex-forming region is strongly associated with nuclease hypersensitive sites and plays a major role in the regulation of Bcl-2 transcription, extensive functional exploration is warranted requiring further investigation [4,5]. Some studies have shown that Bcl-2 is a potent inhibitor of apoptosis. The activation of caspases is a major player and decoder in the process of controlling apoptosis via the regulation of redox equilibrium and disequilibrium, and the fragility of the Bcl-2 major breakpoint region to be cleaved is based upon a non-B-DNA structure [6-9]. In this paper, direct compelling evidence of peroxidase activity by a parallel G-quadruplex-hemin DNAzyme formed by the Pu39WT sequence in the Bcl-2 P1 promoter region is provided, which could help with understanding the complex mechanism of redox equilibrium in the promoter region and its relationship with G-quadruplexes. DNAzymes are catalytically active DNA molecules that can exhibit different enzymatic activities, among which the peroxidase-like activity of some DNA-hemin complexes was discovered [10]. Herein, we demonstrate that Pu39WT incubated with hemin can form a peroxidase-active DNAzyme complex, and that the catalytically active form of Pu39WT is a parallel G-quadruplex conformation rather than a mixed hybrid structure as proposed before. To further exploit the structure–function relationship of the Pu39WT-hemin DNAzyme, the secondary structure and peroxidase activity of Pu39WT-hemin complex were investigated.

## Methods

### Materials and reagents

All experiments were conducted according to the Declaration of Helsinki, and all experimental procedures involving human material were approved by the Institutional Review Board at China Medical University. All oligonucleotides used in this study were synthesized by Sangon Biotechnology Co., Ltd (Shanghai, China). The lyophilized oligonucleotides were dissolved in TE buffer (10 mM Tris–HCl and 0.1 mM EDTA, pH 7.4) to give a stock solution concentration of 100 μM. The experimental concentrations of these oligonucleotides were represented as single-stranded concentrations. Before starting the experiments, the oligonucleotide samples were denatured (5 min at 95°C) to dissociate aggregates and were gradually cooled to room temperature. DMSO, HEPES, Triton X-100 and ABTS were purchased from Sigma-Aldrich and H_2_O_2_ (30%) was from the Beijing Chemical Plant (Beijing, China); all of the chemical reagents were of reagent grade and used without further purification. Hemin was obtained from Sangon Biotechnology Co., Ltd (Shanghai, China). The stock solutions were hemin (100 mM in DMSO), ABTS (100 mM in water), and H_2_O_2_ (60 mM in water). Hemin, ABTS and H_2_O_2_ working solutions were freshly prepared with HEPES buffer (25 mM, pH 7.4) containing 200 mM NaCl, 20 mM KCl, 0.05% Triton X-100, and 1% DMSO before being used. All solutions were prepared with deionized water purified by a Milli-Q system (Millipore, American).

### Circular dichroism (CD) spectroscopy

CD spectra were recorded on a Jasco J-810 spectropolarimeter (Jasco, Easton, MD). 500 μL of sample solution was added to a quartz cell with an optical path length of 1 mm that was placed in a thermostable holder set at 25°C, unless stated otherwise. The CD spectra were representative of three averaged scans at a speed of 500 nm per minute, from 350 nm to 200 nm, with a response time of 1 s and a bandwidth of 2 nm. DNA solutions (DNA at 5 μM, and the DNA-hemin complex at a concentration ratio of 1:1) were prepared in TE buffer (10 mM Tris–HCl and 0.1 mM EDTA, pH 7.4) containing 100 mM KCl or NaCl, and incubated at room temperature overnight to ensure the formation of the quadruplex structure. To facilitate comparisons, the CD spectra were background subtracted, smoothed, and calibrated for concentration so that molar ellipticities could be obtained.

### Ultraviolet visible (UV–vis) study

Absorbance measurements and thermal denaturation experiments were carried out on a Varian Carry 100 (Agilent) UV–vis spectrophotometer equipped with a temperature-control accessory. The solutions were introduced into quartz optical cells with 1 cm optical path-length. Oligonucleotides (DNA at concentration of 5 μM, and the DNA-hemin complex at a concentration ratio of 1:1) were prepared in TE buffer (10 mM Tris–HCl and 0.1 mM EDTA, pH 7.4) containing 100 mM KCl or NaCl. Absorbance spectra were recorded in the 200–350 nm range, with a scan rate of 600 nm per minute and a data interval of 1 nm. UV melting curves of G-quadruplexes or G-quadruplex-hemin (concentration ratio of 1:1) complexes were monitored at 295 nm for absorbance to determine the melting temperature (T_m_). The samples were heated from 15°C to 95°C at a rate of 0.3°C per minute, leaving the samples to equilibrate for 3 min at each temperature before the absorbance signal was recorded.

Thermal difference spectra (TDS) were obtained by simply recording the ultraviolet absorbance spectra of the unfolded and folded states at temperatures above (95°C) and below (15°C) its melting temperature (T_m_), respectively. The difference between these two spectra is the TDS [11].

### Preparation of G-quadruplex-hemin complexes

An equal volume of the 2 × HEPES buffer (50 mM HEPES, 40 mM KCl, 400 mM NaCl, 0.1% Triton X-100, 2% DMSO, pH 7.4) was added to the denatured DNA solutions, and DNA sequences were allowed to properly fold for 40 min. Then, an equivalent amount of hemin in the HEPES buffer (25 mM HEPES, 20 mM KCl, 200 mM NaCl, 0.05% Triton X-100, 1% DMSO, pH 7.4) was added into the above DNA solutions and incubated for 2 h to form G-quadruplex-hemin complexes.

### Peroxidase activity measurements

The color of the reaction mixtures was recorded by a digital camera, and absorption intensity was monitored using a Varian Carry 100 (Agilent) UV–vis spectrophotometer at room temperature. Kinetics were followed by monitoring the appearance of the radical anion (ABTS^•-^) at 421 nm over 80 min for peroxidation catalyzed by either G-quadruplex-hemin (concentration ratio of 1:1) complexes or hemin alone (blank experiments, without G-quadruplex). All kinetic experiments were conducted in quartz optical cells containing buffer, hemin or G-quadruplex-hemin complex, and ABTS. Reactions were initiated by the addition of H_2_O_2_. The final concentrations were as follows: 0.5 μM G-quadruplex, 0.5 μM hemin, 6–18 mM ABTS and 0.6 mM H_2_O_2_. The absorbance intensity at λ = 421 nm was measured as a function of time. The initial rates were calculated from the slope of the initial linear portion (usually the first 20 s) of the increase in absorbance. Kinetic parameters (K_m_ and V_max_) of the peroxidase-catalyzed reactions were obtained using the Michaelis equation.

## Results

### Pu39WT binding hemin formed a uniparallel G-quadruplex structure in K^+^ and Na^+^ solutions, rather than a mixed hybrid structure without bound hemin

**Figure 1:**
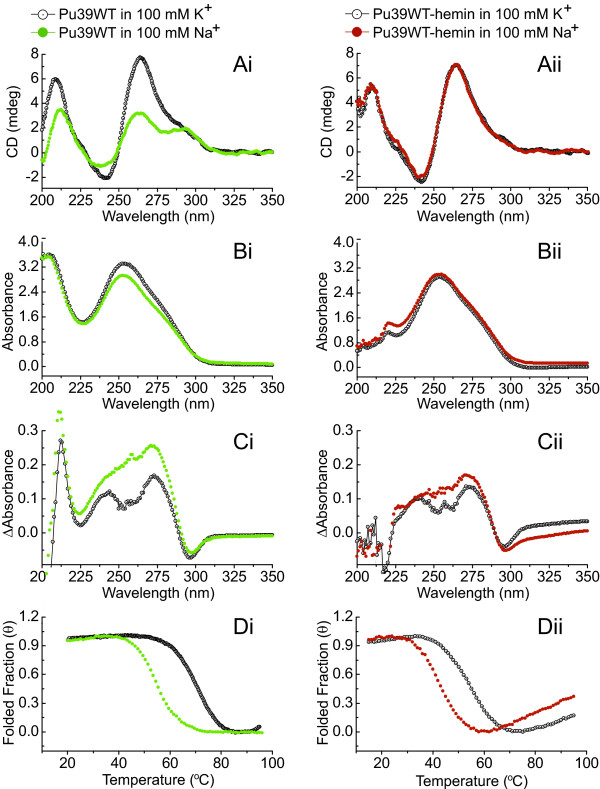
**Structural comparison of Pu39WT G-quadruplex and G-quadruplex-hemin complexes.** Structural comparison of Pu39WT in the absence **(Ai, Bi, Ci)** or presence **(Aii, Bii, Cii)** of hemin (concentration ratio 1:1, at 5 μM DNA concentration). Figures **Di** and **Dii** show melting curves of the Pu39WT G-quadruplex and G-quadruplex-hemin complex, which indicate their values of T_m_ (θ = 0.5).

We compared the structural differences between Pu39WT G-quadruplexes in K^+^ and Na^+^ solution before and after the addition of hemin (Figure 1). Figure 1A shows the CD spectra of the G-quadruplexes, and Figure 1B shows the UV absorbance spectra, both at room temperature. Figure 1C shows the TDS spectra obtained by subtracting the low temperature (15°C) spectra from the high temperature (95°C) spectra (data not shown), with the TDS displayed as the difference between the folded and unfolded forms at temperatures well below and above the corresponding T_m_ (Figure 1D), respectively [11]. Figure 1D shows the normalized folded fraction curves of the Pu39WT G-quadruplex. The folded fraction (θ) was calculated from absorbance versus temperature melting curves, monitored at 295 nm (data not shown), using the conversion of absorbance (A_T_) to θ_T_ at a given temperature by Equation (1):

(1) $$\theta_{T}=\left[A\left(T\right)‒A_{U}\left(T\right)\right]/\left[A_{F}\left(T\right)‒A_{U}\left(T\right)\right]$$

A_F_ and A_U_ represent the upper and lower baselines of the melting curve, respectively [12]. In this representation, a true T_m_ can be determined corresponding to the temperature at which half of the sample is folded and half is unfolded (T_m_ corresponded to θ = 0.5). Corresponding to the results of Dai and Dexheimer et al. [1-3], the CD spectra of Pu39WT displayed an absorption maximum at 264 nm and a minor shoulder at 295 nm, which inferred the formation of a single mixed hybrid G-quadruplex structure [13]. Only the CD amplitudes were smaller in the presence of 100 mM Na^+^ than 100 mM K^+^, which may reflect a decreased tetraplex population (Figure 1Ai). In contrast, after hemin was added, characteristic CD spectra with a positive maximum around 264 nm and a negative minimum around 240 nm were obtained, which indicated a parallel G-quadruplex structure formed by the Pu39WT-hemin complex [13]. Unlike the to hemin-free results, the Pu39WT-hemin CD profiles revealed almost a complete overlap between the spectra in the presence of K^+^ and Na^+^ (Figure 1Aii). At the same time, the UV absorbance spectra of Pu39WT-hemin were also coincident between the K^+^ and Na^+^ environments, compared to Pu39WT without hemin (Figure 1B). From the TDS results (Figure 1C), the major positive peaks around 243 nm and 273 nm indicated the formation of G-quartets, and folded fraction curves (Figure 1D) further demonstrated the formation of a G-quadruplex-hemin complex [11,14]. These data indicate that hemin not only binds Pu39WT to form a Pu39WT-hemin complex, but also, at the same time, alters Pu39WT to a uniparallel G-quadruplex structure from a mixed hybrid one, both in K^+^ and Na^+^ environments.

### Peroxidase activity and detailed parameters of Pu39WT-hemin DNAzyme that govern catalytic efficiency

**Figure 2:**
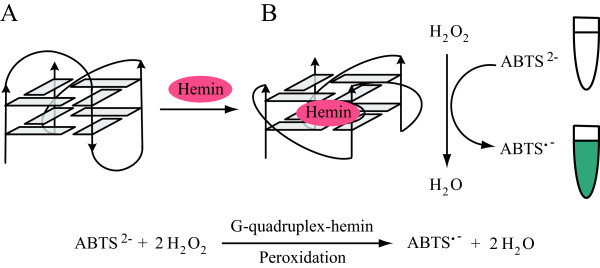
**Diagrammatic rendering of ABTS peroxidation catalyzed by a G-quadruplex-hemin DNAzyme.** This sketch of the G-quadruplex represents Pu39WT, which was induced into a parallel G-quadruplex **(B)** from a mixed hybrid one **(A)** after incubation with hemin.

**Figure 3:**
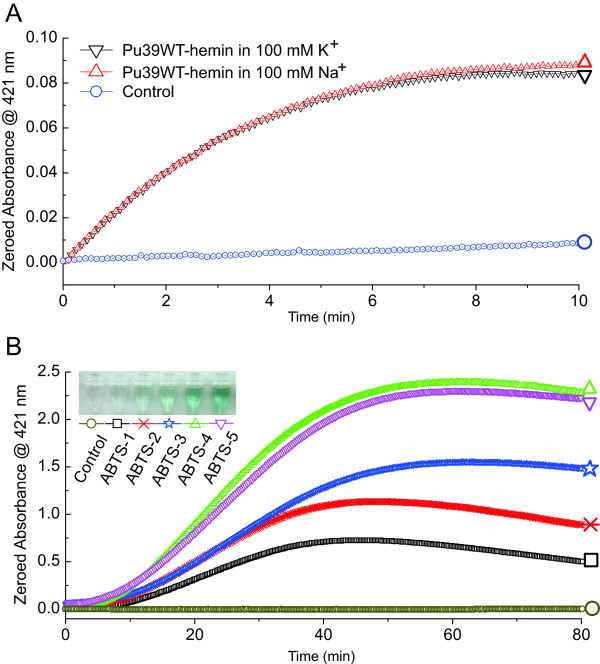
**Peroxidase activity of the Pu39WT-hemin complex. The Pu39WT parallel G-quadruplex-hemin complex has a peroxidase activity. (A)** Comparison of DNAzyme activities of experiments carried out with Pu39WT (0.5 μM), hemin (0.5 μM), H_2_O_2_ (0.6 mM), and ABTS (6 mM) in the presence of either K^+^ or Na^+^. The oxidation of ABTS was monitored over 10 min. **(B)** Catalytic kinetics of the Pu39WT-hemin complex at different substrate (ABTS) concentrations, with Pu39WT (0.5 μM), hemin (0.5 μM), H_2_O_2_ (0.6 mM) and ABTS (from 6 mM to 18 mM). UV–Vis absorbance versus time plots at different substrate concentrations: 6, 9, 12, 15, 18 mM, corresponding to ABTS-1 to 5, respectively; Photograph of visible oxidation of ABTS corresponding to Figure B, taken 40 min after the reaction was triggered.

The peroxidase activities were measured on the basis of the peroxidation of (ABTS^2−^) in the presence of H_2_O_2_ to produce the colored radical anion (ABTS^•-^) (Figure 2), which has a maximal absorption at about 421 nm. Thus, the oxidation of ABTS was monitored via the appearance of the final product-typical UV–vis signal at 421 nm (Figure 3). We drew a comparison between the DNAzyme functions of the Pu39WT-hemin in either K^+^ or Na^+^ in the ABTS-H_2_O_2_ reaction system (Figure 3A). Figure 3A shows that the catalytic activity increased sharply upon Pu39WT incubation with hemin in the ABTS-H_2_O_2_ reaction system (black and red curves), which indicates the formation of a G-quadruplex-hemin DNAzyme. However, Pu39WT formed a parallel G-quadruplex structure, exhibiting strong and almost identical peroxidase activities in K^+^ and Na^+^ environments. Also, from the catalytic kinetics of the G-quadruplex-hemin complex over 10 min (Figure 3A), it can be seen that the DNAzyme function of Pu39WT-hemin is almost independent of a K^+^ or Na^+^ ion effect. That is, once the DNAzyme was formed by Pu39WT binging hemin, it possessed a stable peroxidase activity. These results indicated that Pu39WT-formed G-quadruplex-hemin complexes, in K^+^ and Na^+^, possess a stable general peroxidase activity and the intramolecular parallel G-quadruplex structure may be essential for catalysis.

**Figure 4:**
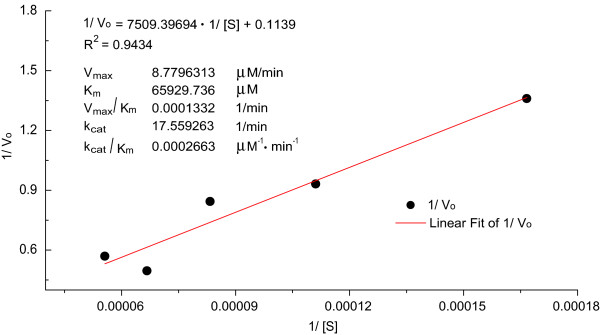
**Lineweaver-Burk plot and obtained detailed DNAzyme catalytic parameters.** The k_cat_ (min^−1^) value was obtained from the equation: k_cat_ = V_max_/[E]. All the kinetic measurements were repeated at least three times and were found to agree within ±5-10%.

To evaluate this peroxidase-like catalytic activity of Pu39WT-hemin, a catalytic reaction was carried out with 0.5 μM Pu39WT-hemin DNAzyme in 6, 9, 12, 15 and 18 mM ABTS and 0.6 mM H_2_O_2_. Figure 3B shows absorbance at 421 nm versus time plots for different ABTS concentrations indicating peroxidation by Pu39WT-hemin. As the reaction progress was monitored using UV–vis spectroscopy at 421 nm in kinetic mode to measure the reaction rate V_0_, it followed a conventional enzymatic dynamic regulation reflected in the Michaelis-Menten equation. The Michaelis constant K_m_ and catalytic constant k_cat_ were calculated by the Lineweaver-Burk plot (see Equation (2)), as shown in Figure 4:

(2) $$1/V=K_{m}/V_{\text{max}}1/\left[S\right]+1/V_{\text{max}}$$

From the Lineweaver-Burk plot, the Michaelis constant K_m_ and the catalytic constant k_cat_ of Pu39WT-hemin in the ABTS oxidation reaction were calculated to be 65.929736 mM and 17.559263 min^−1^ (Figure 4), indicating a high DNAzyme affinity and catalytic activity, respectively. In addition, its catalytic efficiency (k_cat_/K_m_) was calculated. Of the Pu39WT oligonucleotides, the parallel G-quadruplex-hemin complex showed such obviously high and visible peroxidase activity that the reaction mixture produced a characteristic green color (Figure 3B). All of these data reveal the detailed parameters and powerful peroxidase activity properties of Pu39WT-hemin DNAzyme.

## Discussion

Although DNAzymes have garnered much interest because of their virtually unlimited applications in developing new molecular devices in biodetection, biosensing and bioanalysis, some researchers have focused their attention on structure–function studies of genetic sequences and the molecular mechanisms of disease. Recent studies have shown the DNAzyme properties of human telomeric DNA sequences [15,16], and general peroxidase activity of several G-quadruplex-forming sequences in human gene promoters (including a partial sequence of Bcl-2, Pu39WT) [17,18]. In this study, we report for the first time that Bcl-2 Pu39WT, a mixed hybrid G-quadruplex forming sequence reported previously, can form a parallel G-quadruplex-hemin DNAzyme displaying stable peroxidase activity in either K^+^ or Na^+^ ionic conditions. Our results indicate that: 1, hemin can not only bind Pu39WT to form a Pu39WT-hemin complex, but also alters Pu39WT into a uniparallel G-quadruplex structure from a mixed hybrid one, in both K^+^ and Na^+^ environments; and 2, the Pu39WT-formed parallel G-quadruplex-hemin DNAzyme possesses a stable and powerful peroxidase activity with detailed parameters that govern the catalytic efficiency.

The observed results not only serve to acquaint us with the possible role of the Pu39WT G-quadruplex as a DNAzyme ingredient, but also provide us with insight into how gene promoter regions with G-quadruplex-forming sequences might be related to actual DNAzyme catalytic reactions *in vivo* (which still remains a question that is open to debate). They likewise cast new light on the importance of G-quadruplexes in potential hemin-mediated mechanisms of cellular injury and tumorigenesis, which may open up a new avenue for gene therapy.

## Conclusion

Our results showed the general peroxidase activity of Pu39WT-hemin DNAzyme, the intramolecular parallel G-quadruplex structure of which favors the progression of peroxidase activity and presents a high peroxidase activity. This peroxidase activity of the hemin complexed with a G-quadruplex-forming sequence in the Bcl-2 gene promoter may imply a potential mechanism of hemin-mediated cellular injury. This structure-dependent peroxidase activity has also been demonstrated to be applicable for colorimetric screening of G-quadruplex ligands.

## Abbreviations

DMSO: Dimethylsulfoxide; HEPES: N-2-hydroxyethylpiperazine-N’-2-ethanesulfonicacid; ABTS: 2,2’-amino-di (2-ethyl-benzothiazoline sulphonic acid-6) ammonium salt; H_2_O_2_: Hydrogen peroxide; CD: Circular dichroism; UV: Ultraviolet; TE: Tris–HCl EDTA; EDTA: Ethylene Diamine Tetraacetic Acid; T_m_: Melting temperature; TDS: Thermal difference spectrum; K_m_: Michaelis constant; k_cat_: Catalytic constant; V_max_: The maximum velocity.

## Competing interests

The authors declare that they have no competing interests.

## Authors’ contributions

BL and HS conceived of the study, participated in its design and drafted the manuscript. BL and DL carried out data acquisition, interpretation and performed the statistical analysis. All authors read and approved the final manuscript.
