# Erratum: General peroxidase activity of a parallel G-quadruplex-hemin DNAzyme formed by Pu39WT - a mixed G-quadruplex forming sequence in the Bcl-2 P1 promoter

**DOI:** 10.1186/s13065-014-0057-0

**Published:** 2014-09-30

**Authors:** Bo Liu, Hong Shang, Da Li

**Affiliations:** Department of Laboratory Medicine, the First Hospital of China Medical University, Shenyang, 110001 China

## Abstract

This is a correction to the following paper: General peroxidase activity of a parallel G-quadruplex-hemin DNAzyme formed by Pu39WT - a mixed G-quadruplex forming sequence in the Bcl-2 P1 promoter, Bo Liu, Da Li* and Hong Shang*, Chemistry Central Journal 2014, 8:43 (1 July 2014).

## Correction

This is a correction to the following paper: General peroxidase activity of a parallel G-quadruplex-hemin DNAzyme formed by Pu39WT - a mixed G-quadruplex forming sequence in the Bcl-2 P1 promoter, Bo Liu, Da Li* and Hong Shang*, Chemistry Central Journal 2014, 8:43 (1 July 2014) [1].

Authors have requested a change in order of the corresponding authors.
